# Tryptophan metabolism and erectile dysfunction: pathophysiological insights into kynurenine pathway dysregulation

**DOI:** 10.1186/s12894-026-02312-0

**Published:** 2026-08-13

**Authors:** Muammer Özdemi̇r, Şaban Kadir Köleli̇, Ali Ünlü, Mohammad Ahmad Bi̇k, Murat Gül

**Affiliations:** 1 Department of Medical Biochemistry, Karamanoğlu Mehmetbey University Medical Faculty, Karaman, Türkiye; 2 Department of Urology, Selçuk University Medical Faculty, Konya, Türkiye; 3 Department of Medical Biochemistry, Selçuk University Medical Faculty, Konya, Türkiye

## Abstract

**Background:**

ED refers to the persistent difficulty in attaining or sustaining a firm enough erection for satisfactory sexual performance. The kynurenine pathway (KP) of tryptophan metabolism is associated with reactive oxygen species production and inflammation and may be related to the pathophysiology of ED. This study aimed to analyze KP biomarker levels in ED patients and examine their potential connection to ED.

**Methods:**

110 consecutive male patients diagnosed with ED and 60 age-matched healthy male volunteers were included. Serum KP metabolite levels were analysed using the liquid chromatography tandem mass spectrometry method (LC-MS/MS). Multivariate Firth penalized logistic regression was used to assess the independence of metabolite associations, adjusted for age, BMI, total testosterone, LH, FSH, prolactin and smoking status.

**Results:**

Compared to the control group, ED patients showed significantly lower TRP (6,760 vs 10,600 ng/mL, p < 0.001) and KYNA levels (1.39 vs 2.19 ng/mL, p < 0.001), while KYN, 3-HK, 3-HAA and QA levels were significantly higher (p < 0.001 for all). In Firth penalized logistic regression adjusted for age, BMI, smoking status, total testosterone, LH, FSH and prolactin, all metabolite associations remained independently significant (e.g., 3-HK OR 13.44, 95% CI 5.02–35.99; TRP OR 0.24, 95% CI 0.14–0.43; all q < 0.05). In exploratory ROC analysis, 3-HK (apparent AUC 0.902; LOOCV AUC 0.898) and TRP (AUC 0.811; LOOCV 0.801) revealed the highest performance; according to the DeLong test, the AUC of 3-HK was significantly higher than that of TRP (Z = 2.295, p = 0.021). In the subgroup analysis of severity, QA levels were similar between the control and mild ED groups, while they were significantly increased in moderate to severe cases.

**Conclusions:**

These findings suggest an independent association between ED and KP metabolite imbalance, highlighting the kynurenine pathway as a potential target for future research.

## Introduction

ED refers to the persistent difficulty in attaining or sustaining a firm enough erection for satisfactory sexual performance and is a prevalent issue that greatly impacts men’s quality of life [1]. The prevalence of ED is estimated to affect approximately 40% of men at the age of 40, rising to nearly 70% by the age of 70 [2]. Importantly, ED is not only a disorder of sexual function but is also considered an early marker of systemic diseases such as cardiovascular disease, diabetes, neurological disorders and psychiatric illnesses [3].

The etiopathogenesis of ED is multifactorial, including vascular, neurological, hormonal, psychological and pharmacological causes. In recent years, biochemical processes such as oxidative stress, inflammation and neurotransmitter imbalances have also been shown to play crucial roles in the development of ED [4].

Neurotransmitter systems such as serotonin, dopamine and nitric oxide are central to the physiology of erection. In particular, central serotonergic inhibition has been associated with decreased libido and erectile impairment [5]. In this context, the metabolism of tryptophan, a serotonin precursor, has become an important area of research in understanding ED pathophysiology.

Tryptophan metabolism includes both the indole and kynurenine pathways. A careful equilibrium exists among the various tryptophan pathways. Current understanding indicates that roughly 95% is processed through the kynurenine pathway. Since the serotonin and kynurenine metabolic pathways compete for the same substrate: tryptophan, an increased metabolism through the kynurenine pathway may lead to a relative shortage of serotonin in the central nervous system [6].

**Fig. 1:**
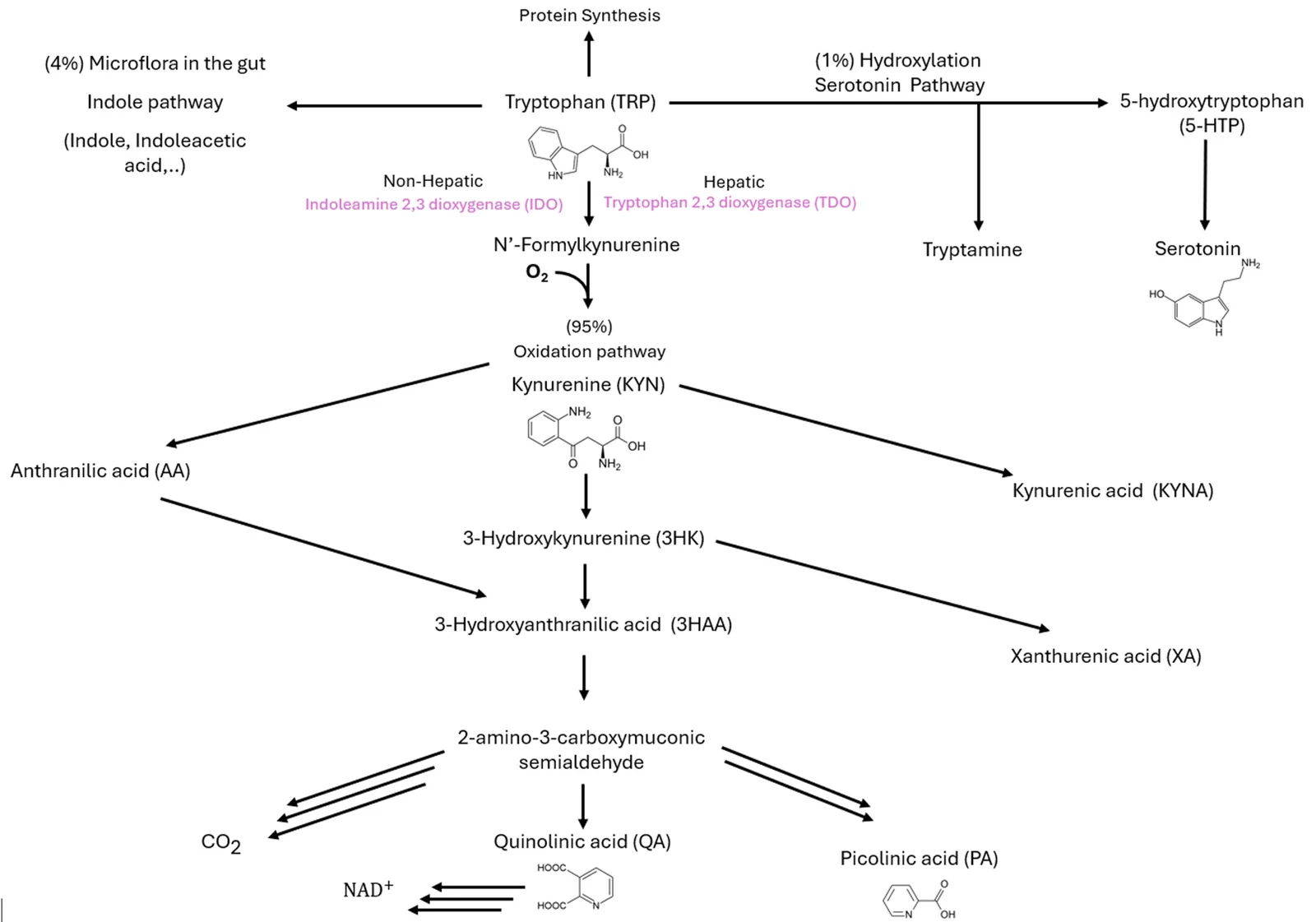
Trypophan metabolism. TRP follows 4 main routes: Kynurenine, serotonine, indole (gut microbiota) and protein synthesis pathways [8].

Nearly 1–2% of tryptophan intake transforms to the serotonin and the primary pathway for the metabolism of tryptophan is the kynurenine pathway, which 95% of tryptophan is oxidised into biologically active molecules that affect multiple pathways, like ROS generation by activation of indolamine dioxygenase (IDO) and synthesis of 3-OH Kynurenine (3-HK) and 3-OH Anthranilic acid (3-HAA), also glutamate receptors, by the formation of kynurenic acid and quinolinic acid. These compounds can have different effects on several receptors: kynurenine (KYN) and kynurenic acid (KYNA) which act as agonists at Aryl hydrocarbon receptors (AhR). While KYNA has antagonising effects for the N-methyl D-aspartate (NMDA) receptor family, quinolinic acid (QA) acts as an agonist for the NMDA receptor family. According to the existing literature, 3-HK, 3-HAA and QA are related to ROS generation [7]. The main metabolic pathways of tryptophan degradation, including the kynurenine pathway, are schematically depicted in Fig. 1.

In this study, we used the liquid chromatography tandem mass spectrometry method (LC-MS/MS) to analyse KP biomarker levels in ED patients and examine their potential connection to erectile dysfunction.

## Materials and methods

### Study design and participants

This prospective and observational study was conducted at the Urology Department of Selcuk University from February 2024 to January 2025. 110 consecutive male patients aged 30–65 years diagnosed with erectile dysfunction (ED) were included, along with 60 age-matched healthy male volunteers serving as the control group. Approval for the study protocol was granted by the Ethics Committee of Selcuk University, Faculty of Medicine (Approval No: 2024/67). Every participant gave their written informed consent before inclusion.

### Inclusion and exclusion criteria

Clinical ED diagnosis was based on an IIEF-5, a previously validated and published questionnaire [9], using a cutoff score of ≤ 21. Inclusion criteria required a history of ED for at least 6 months and no use of medications affecting sexual function in the previous 4 weeks. All study participants declared that they participated in sexual activity on a regular basis (minimum of once a week) with a stable heterosexual partner.

Exclusion criteria included known cardiovascular, endocrine, hepatic, renal, neurological, or psychiatric diseases, active infections or malignancies, or a history of pelvic surgery. The control group consisted of men without ED (IIEF-5 ≥ 22), with no relevant systemic illness or sexual dysfunction.

### Severity grouping and questionnaire

ED patients were categorized into 3 subgroups based on IIEF-5 scores: mild (12–21), Moderate (8–11) and Severe (5-7). Both questionnaire were administered in a private clinical setting by trained staff during outpatient visits.

### Study process

Body mass index (BMI) is determined by calculating values from the recorded height and weight. Following the physical examination, participants underwent an overnight fasting period. A blood sample of 10 mL was collected from the antecubital vein the next morning, between 8 and 10 AM, into both BD (Becton-Dickinson, Franklin Lakes, NJ, USA) Vacutainer Serum Separator Tubes (SST) and BD Vacutainer^®^ EDTA Tubes. Fasting serum samples were collected and stored at − 80 °C until analysis. Serum samples were collected in Eppendorf tubes, protected from light and stored at -80 °C until analysis. On the day of analysis, serum samples were equilibrated to room temperature at the Selçuk University Faculty of Medicine Hospital Metabolism Laboratory.

Concentrations of tryptophan and its metabolites were measured by the LC-MS/MS system used was Sciex API 3200 (triple quadrupole mass spectrometer) and the method that was found from Abusoglu et al. used for the analysis of the metabolites. The A gradient mobile phase was used in the study. HPLC-grade water containing 0.1% formic acid was used in mobile phase A, while acetonitrile containing 0.1% formic acid was used in mobile phase B. The prepared mobile phases were vortexed and incubated in an ultrasonic bath for 30 min at room temperature to ensure homogeneity. The precipitant consisted of acetonitrile with 1% formic acid, while the solvent was prepared as a mixture of 25% acetonitrile and 75% grade water, both containing 1% formic acid.

Following the thawing of serum samples at room temperature, 300 µL of each sample was spiked with 100 µL of internal standard. Subsequently, 1000 µL of acetonitrile (containing 1% formic acid) was added and the mixture was rigorously vortexed for 30 s prior to analysis. The sample was then centrifuged at 12,000 rpm for 10 min. The supernatant was transferred to a clean glass tube and dried under nitrogen gas at room temperature. After drying, the solution was dissolved in 250 µL of 0.1% formic acid (25% acetonitrile and 75% water) and transferred to the vials.

The instrument specifications used for the chromatographic separation for the analysis of TRP and its metabolites in serum are: The mobile phase was set as the gradient. Mobile phase A: 100% HPLC grade water containing 0.1% formic acid. Mobile phase B: 100% acetonitrile containing 0.1% formic acid. The flow rate was set at 1 mL/min and the analysis time for each sample was 5 min. The autosampler temperature was set at 4 °C and the column temperature was set at 25 °C. Injection volume: 40 µL Positive ion electrospray ionization (ESI) SRM mode was used. Ion-spray voltage: 3000 V. Heater temperature: 450 psi, Curtain gas: 15 psi [10].

### Statistical analysis

Statistical evaluations were executed utilizing Python software (version 3.11). The Kolmogorov-Smirnov test was employed to evaluate the normality of continuous data. Since the variables exhibited a non-normal distribution, results were expressed as medians along with their respective ranges (minimum–maximum). For intergroup comparisons of independent samples, the Mann-Whitney U and Kruskal-Wallis tests were applied, the latter being followed by Bonferroni-corrected post-hoc analyses.

Multiple testing correction was performed using the False Discovery Rate (FDR) approach. Adjusted p-values (q-values) less than 0.05 were considered statistically significant. To test the model’s stability, a leave one out (LOO) cross-validation method was used as: each comparison was re-run after removing the single lowest and single highest value and a finding was considered robust when its FDR-adjusted significance (q < 0.05) was preserved, the recomputed p-values are reported. Bivariate correlations were investigated by Pearson’s test or Spearman’s test (whether the distribution was normal or not).

Receiver Operating Characteristic (ROC) curve analysis was used to perform an exploratory evaluation of the discriminatory ability of the tested parameters. The internal validity of ROC models was assessed using leave-one-out cross-validation and bootstrap optimism correction (Harrell method, 1000 replicates); cross-validated AUC, sensitivity, specificity, PPV, NPV and 95% confidence intervals were reported. Optimal cut-off values were determined based on the Youden index. Values under *p* < 0.05 were considered statistically significant.

Multivariate logistic regression was applied to assess the independence of metabolite relationships. Each kynurenine pathway metabolite was included as an independent variable in a separate model adjusted for variables identified as potential confounders (age, BMI, total testosterone, LH, FSH, prolactin and smoking status). Given the problem of multicollinearity and potential separation between metabolites, Firth penalized logistic regression, which provides unbiased estimation in small/divergent data was used. Since continuous variables were standardized, odds ratios (ORs) were given per 1 standard deviation (SD). Multicollinearity was assessed using the variance inflation factor (VIF).

## Results

**Fig. 2:**
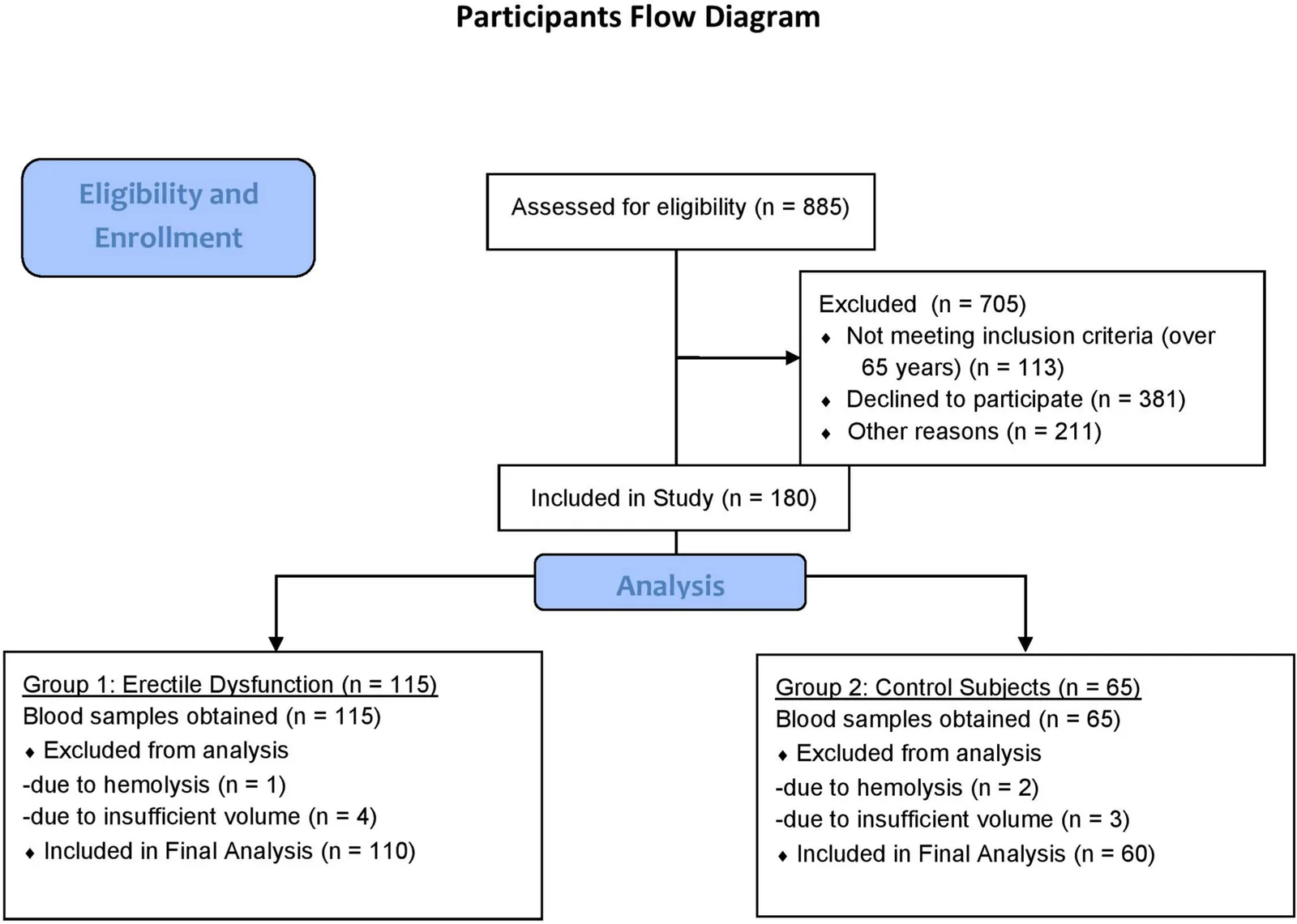
Participants flow diagram

The study collected data from 110 ED patients and 60 healthy controls. The groups exhibited comparable average ages and BMI distributions (*p* = 0.811 and *p* = 0.301, respectively). Patients with ED had significantly lower levels of TRP and KYNA, while levels of KYN, 3-HK, 3-HAA and QA were significantly higher. (Fig. 2; Table 1).

**Table 1:** Demographic, clinical and laboratory characteristics of the participants

	ALL ED Patients (*n* = 110)	Controls (*n* = 60)	*p*-value	q-value	Cohen’s d	LOO *p* (min-max)
Demographical characteristics						
Age (Year)	50,50 [37,25–62]	50 [31–66]	0,811ᵃ	0,857	0.07	0,953-0,670
Body Mass Index (BMI)	27,07 [24,87–29,09]	26,89 [24,69–28,73]	0,301ᵃ	0,335	0.28	0,250 − 0,349
Smoking Status	29 (39,2%)	25 (47,2%)	0,238ᵇ	0,335		-
Packs per day	0 [0–1,50]^c^	0 [0–2]^c^	0,293ᵃ	0,335	0.21	0,322 − 0,390
Laboratory Parameters						
Total Testosterone	411 [301,50–510,50]	427 [342,50–526,75]	0,555ᵃ	0,629	-0.14	0,639-0,468
Estradiol	24,95 [18,23–31,90]	24 [19,25–30,85]	0,857ᵃ	0,857	0.00	0,754 − 0,960
Prolactin	10,90 [8,26–13,60]	10,30 [7,08–13,07]	0,158ᵃ	0,269	0.32	0,215-0,195
FSH	4,91 [3,26–7,48]	5,06 [3,97–8,18]	0,284ᵃ	0,366	-0.22	0,341 − 0,372
LH	5,76 [4,21–8,17]	6,87 [4,91–8,56]	0,094ᵃ	0,178	-0.28	0,118-0,069
Tryptophan (ng/mL)	6,760 [4960–8600]	10,600 [9200–12300]	**< 0**,**001ᵃ**	**< 0**,**001**	-1.25	**< 0**,**001-<0**,**001**,
KYN (ng/mL)	328 [196–524]	231 [190–336]	**0**,**021ᵃ**	**0**,**046**	0.53	**0**,**016 − 0**,**026**,
Kyn to Trp Ratio (KTR)	0,05 [0,03–0,07]	0,03 [0,02–0,04]	**< 0**,**001ᵃ**	**< 0**,**001**	0.86	**< 0**,**001-<0**,**001**,
3-HK (ng/mL)	8,29 [6,30–10,45]	2,60 [1,51–5,16]	**< 0**,**001ᵃ**	**< 0**,**001**	1.56	**< 0**,**001-<0**,**001**,
3-HK to KYN Ratio	0,03 [0,01–0,04]	0,01 [0,01–0,02]	**< 0**,**001ᵃ**	**< 0**,**001**	0.90	**< 0**,**001-<0**,**001**,
3-HAA (ng/mL)	5,88 [3,36–8,69]	3,48 [1,86–5,27]	**< 0**,**001ᵃ**	**< 0**,**001**	0.76	**< 0**,**001-<0**,**001**,
KYNA (ng/mL)	1,39 [0,74–2,21]	2,19 [1,39–2,87]	**< 0**,**001ᵃ**	**< 0**,**001**	-0.56	**< 0**,**001-<0**,**001**,
QA (ng/ml)	4,60 [2,70–5,70]	2,60 [1,80–3,30]	**< 0**,**001ᵃ**	**< 0**,**001**	-0.99	**< 0**,**001-<0**,**001**,

ᵃ=Mann Whitney U, ^b^=Chi Square with Yates test, ^c=^given as min-max, the rest are given as IQR 25th -75th percentile. Bold values indicate statistically significant results

Demographic, clinical and laboratory data of the included individuals are listed in Table 1.

**Fig. 3:**
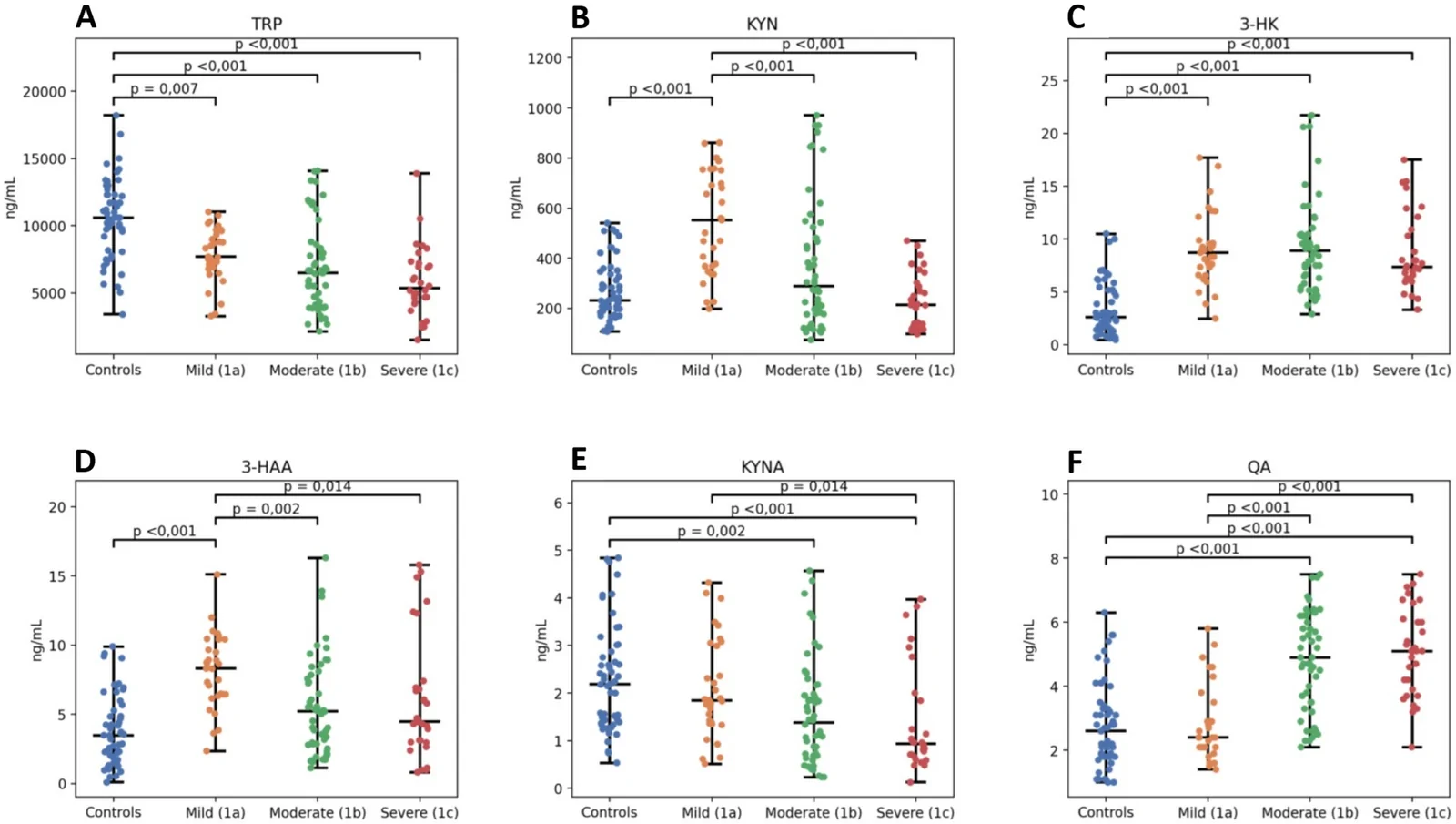
The serum level of (**A**) TRP (ng/mL), (**B**) KYN (ng/mL), (**C**) 3-HK (ng/mL), (**D**) 3-HAA (ng/mL), (**E**) KYNA (ng/mL) and (F) QA (ng/mL) between the groups assessed by IIEF-5. The black line represents median and min-max levels, dots represent individual values

The subgroup distribution of ED patients according to their IIEF-5 scores was as follows: mild (12–21) n = 30, moderate (8–11) n = 50, severe (5–7) *n* = 30. In the intergroup comparison, TRP, KYN, KTR, 3-HK, 3-HK/KYN ratio, 3-HAA, KYNA and QA levels revealed significant differences between groups (q < 0.001 for all; Table 2). Particularly noteworthy was that KYN concentrations reached their highest levels in the mild ED group and decreased relative to the moderate and severe groups, while QA levels remained similar between the control and mild groups and increased significantly in the moderate and severe groups (Table 2; Fig. 3). Age and LH distributions showed nominal differences between groups but did not reach the significance limit after FDR correction (q = 0.061 and q = 0.061, respectively). A detailed breakdown of demographic and clinical characteristics by subgroup is presented in Table 2.

**Table 2:** Comparison of demographic, clinical and laboratory characteristics across ED severity subgroups and controls

	Controls (*n* = 60)	Mild (*n* = 30)	Moderate (*n* = 50)	Severe (*n* = 30)	*p*-value	q-value (FDR)	Epsilon-sq	LOO *p* (min-max)
*Demographical characteristics*								
*Age (year)*	50 [31–66]	44,50 [35,75–48,25]	49 [35–62]	63 [52,75–69,50]	0,034ᵃ	0,061	0.069	0,752-0,987
*Body mass index (BMI)*	26,89 [24,69–28,73]	28 [24,93–31]	27,72 [25,13–29,39]	25,47 [24,49–27,18]	0,236ᵃ	0,334	0.029	0,818-0,094
*Smoking status*	25 (47,2%)	9 (29,0%)	22 (42,3%)	3 (25,0%)	0,269ᵇ	0,352	-	-
*Packs per day*	0 [0–2]^c^	0 [0–1,5]^c^	0 [0–1,5]^c^	0 [0–1,5]^c^	0,543ᵃ	0,616	0.017	0,514-0,683
*Laboratory parameters*								
*Total testosterone*	427 [342,50–526,75]	428,50 [357,50–519,50]	435 [244–532]	383 [353,25–478,25]	0,824ᵃ	0,876	0.007	0,592-0,381
*Estradiol*	24 [19,25–30,85]	24,90 [19,35–32,58]	24,95 [16,12–31,95]	24,25 [21,50–31,50]	0,956ᵃ	0,956	0.003	0,916-0,812
*Prolactin*	10,30 [7,08–13,07]	10,90 [8,70–11,93]	11,10 [8,28–13,90]	10,80 [9,59–16,35]	0,542ᵃ	0,616	0.018	0,786-0,673
*FSH*	5,06 [3,97–8,18]	4,65 [4,08–5,49]	4,58 [2,75–7,47]	6,50 [5,09–13,40]	0,085ᵃ	0,131	0.055	0,113-0,114
*LH*	6,87 [4,91–8,56]	5,39 [4,34–6,81]	5,45 [4,18–7,84]	9,15 [5,53–10,82]	**0**,**036ᵃ**	0,061	0.071	0,569-0,953
*Tryptophan (ng/ml)*	10,600 [9200–12300]	7698 [6843,50–9463,50]	6480 [4085–8390]	5355 [4392,50–7154]	**< 0**,**001ᵃ**	**< 0**,**001**	0.294	0,337-0,234
*KYN (ng/ml)*	231 [190–336]	552 [368,50–724,50]	288 [178,50–491,75]	214,50 [130,25–298,25]	**< 0**,**001ᵃ**	**< 0**,**001**	0.224	0,020 − 0,042
*KTR*	0,03 [0,02–0,04]	0,07 [0,05–0,09]	0,05 [0,03–0,07]	0,04 [0,02–0,06]	**< 0**,**001ᵃ**	**< 0**,**001**	0.300	**<0**,**001 − 0**,**001**
*3-HK (ng/ml)*	2,60 [1,51–5,16]	8,71 [6,96–9,74]	8,90 [6,17–10,45]	7,38 [6,26–10,75]	**< 0**,**001ᵃ**	**< 0**,**001**	0.425	**<0**,**001 − 0**,**001**
*3-HK to KYN ratio*	0,01 [0,01–0,02]	0,02 [0,01–0,03]	0,03 [0,01–0,05]	0,04 [0,02–0,06]	**< 0**,**001ᵃ**	**< 0**,**001**	0.324	**0**,**011 − 0**,**008**
*3-HAA (ng/ml)*	3,48 [1,86–5,27]	8,30 [6,44–10,04]	5,23 [2,83–7,64]	4,48 [3,02–6,90]	**< 0**,**001ᵃ**	**< 0**,**001**	0.203	**0**,**035 − 0**,**027**
*KYNA (ng/ml)*	2,19 [1,39–2,87]	1,85 [1,38–3,02]	1,39 [0,68–2,03]	0,94 [0,71–1,69]	**< 0**,**001ᵃ**	**< 0**,**001**	0.142	0,525-0,384
*QA (ng/ml)*	2,60 [1,80–3,30]	2,40 [2,10–3,20]	4,90 [3,50–6,20]	5,10 [3,98–6,07]	**< 0**,**001ᵃ**	**< 0**,**001**	0.385	0,777-0,673

ᵃ=Kruskal Wallis test with Bonferroni correction, ^b^=Chi Square with Yates test, ^c=^given as min-max, the rest are given as IQR 25th -75th percentile. Bold values indicate statistically significant results

**Fig. 4:**
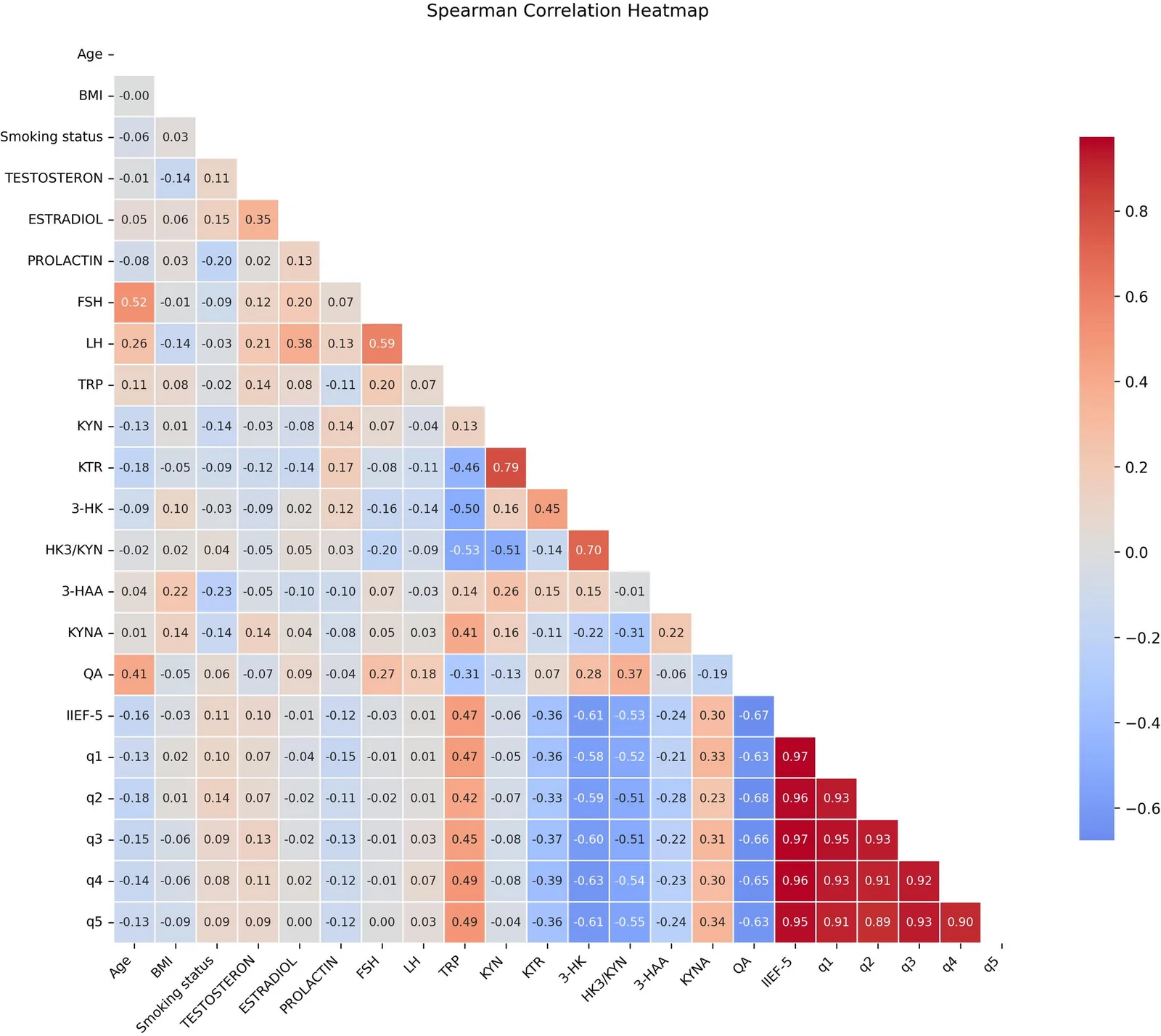
Correlation Heatmap diagram revealing the relationship between demographic and laboratory values. Coefficients were represented by colored gradation

Spearman’s rho correlation analysis demonstrated significant associations between kynurenine pathway metabolites and IIEF-5 scores. Specifically, a statistically significant positive correlation was observed for TRP (rho = 0.465, *p* < 0.001) and KYNA (rho = 0.300, *p* < 0.001). Conversely, significant negative associations were identified for 3-HK (rho=-0.608, *p* < 0.001), 3-HAA (rho=-0.244, *p* = 0.005) and QA (rho=-0.670, *p* < 0.001). No significant correlation was found between KYN levels and IIEF-5 scores (rho=-0.064, *p* = 0.476). The correlation heatmap/matrix illustrating the relationships among measured metabolites is shown in Fig. 4.

After adjusting for age, BMI, total testosterone, LH, FSH, prolactin and smoking status, all metabolite associations remained independently significant (Table 3). While decreased TRP was inversely associated with ED (OR 0.24 per SD; 95% CI 0.14–0.43), increases in 3-HK (OR 13.44; 5.02–35.99), QA (OR 11.29; 4.62–27.59) and KTR (OR 8.42; 2.79–25.44) were strongly and independently associated with ED (q < 0.05 for all). All variance inflation factors were below 5 (< 1.7), indicating no significant multicollinearity in the models. Multivariate regression analyses results are listed in Table 3.

**Table 3:** Multivariate regression analysis

Metabolite	Adjusted OR	95% CI	*p*	q (FDR)	VIF
TRP	0.24	0.14–0.43	**< 0.001**	**< 0.001**	3.00
KYN	1.63	1.04–2.55	**0.033**	**0.033**	2.76
KTR	8.42	2.79–25.44	**< 0.001**	**< 0.001**	4.12
3-HK	13.44	5.02–35.99	**< 0.001**	**< 0.001**	1.78
3-HAA	2.47	1.43–4.27	**0.001**	**0.002**	1.18
KYNA	0.51	0.33–0.78	**0.002**	**0.002**	1.21
QA	11.29	4.62–27.59	**< 0.001**	**< 0.001**	1.80

Estimator: Firth penalized. Adjusted for: Age, BMI, Total Testesteron, LH, FSH, Prolactin, Smoking Status. Bold values indicate statistically significant results

**Fig. 5:**
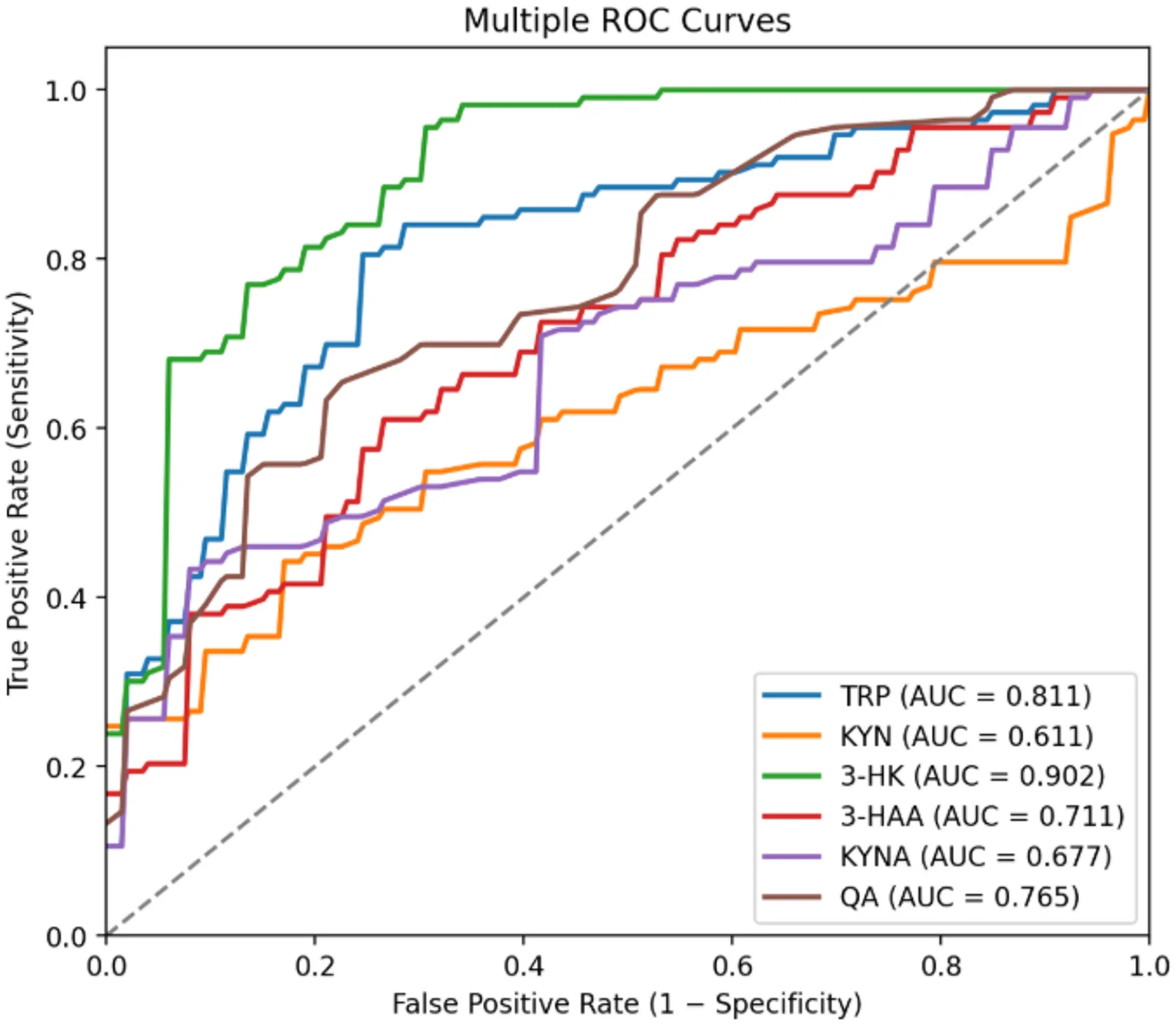
Exploratory ROC analysis results of the laboratory parameters for the discrimination of ED

ROC analysis was used to exploratorily evaluate the discriminatory ability of individual laboratory parameters for ED patients in an exploratory framework. The ROC curve analysis revealed that the Area Under the Curve (AUC) values for the discrimination of ED were 0.811 (95% CI, 0.734–0.877; *p* < 0.001) for TRP, 0.611 (95% CI, 0.527–0.692; *p* = 0.0214) for KYN, 0.902 (95% CI, 0.846–0.951; *p* < 0.001) for 3-HK, 0.711 (95% CI, 0.629–0.788; *p* < 0.001) for 3-HAA, 0.677 (95% CI, 0.595–0.752; *p* < 0.001) for KYNA and 0.765 (95% CI, 0.684–0.830; *p* < 0.001) for QA (Table 4 and Fig. 5). 

**Table 4:** ROC analysis with internal validation

Marker	AUC (apparent)		95% CI	AUC (LOOCV)	AUC (optimism-corr.)	Sensitivity	Specificity	PPV	NPV
*TRP*	0.811	0.741–0.881		0.801	0.812	0.805	0.755	0.875	0.645
*KYN*	0.611	0.527–0.695		0.583	0.611	0.442	0.830	0.847	0.411
*3-HK*	0.902	0.849–0.955		0.898	0.903	0.956	0.698	0.871	0.881
*3-HAA*	0.711	0.629–0.793		0.693	0.710	0.611	0.736	0.831	0.470
*KYNA*	0.677	0.595–0.759		0.655	0.678	0.434	0.925	0.925	0.434
*QA*	0.765	0.690–0.841		0.753	0.766	0.655	0.774	0.860	0.512

## Discussion

In this research, we aimed to evaluate the levels of specific tryptophan metabolites on individuals with erectile dysfunction and to investigate their possible associations with ED. The results indicate significant changes in all KP metabolites among individuals with varying degrees of ED severity and these differences are statistically significant in age and BMI-matched healthy individuals. These associations remained independent even after multivariate adjustment (Table 3). Among the KP metabolites, TRP and 3-HK showed the highest AUCs in the exploratory single-marker ROC analysis.

Previous studies have suggested that IFN-γ accelerates the conversion of tryptophan to kynurenine by stimulating the IDO enzyme. In our study, the significantly higher levels of KYN and KTR in the ED group compared to controls (KYN: 328 vs. 231 ng/mL, q = 0.046; KTR: 0.05 vs. 0.03, *p* < 0.001) and significantly lower levels of TRP (6,760 vs. 10,600 ng/mL, *p* < 0.001) suggest that the kynurenine pathway is regulated differently in ED. Moreover, IIEF-5 scores revealed a positive correlation with TRP and negative correlation with KTR. This metabolic profile is consistent with previous findings showing elevated IFN-γ response chemokines in ED [11]. Whether this metabolic shift reflects IFN-γ–driven IDO activation, as suggested by the literature, remains to be confirmed through direct enzymatic and cytokine measurements.

Inter-group analyses revealed distinct metabolic profiles corresponding to ED severity. Following post-hoc correction, TRP levels were found to be significantly depleted in all ED subgroups compared to healthy controls. Interestingly, KYN concentrations peaked in the mild ED group but exhibited a relative decline in moderate and severe cases. Similarly, the KTR was highest in mild ED patients compared to the other three groups (*p* < 0.001 vs. controls; *p* = 0.064 vs. moderate; *p* < 0.005 vs. severe cases). While this nonlinear pattern suggests dynamic metabolic shifts across severity stages, whether these variations reflect temporal disease progression or distinct pathophysiological subtypes warrants prospective evaluation. The detailed distribution of metabolite levels among severity groups is presented in Table 2.

KYN and KYNA are natural ligands of the aryl hydrocarbon receptor (AhR); AhR is notable for its role in androgen receptor modulation and steroidogenesis in male reproductive health [12, 13]. In our study, KYNA levels were found to be significantly lower in the ED group compared to controls (1.39 vs. 2.19 ng/mL, *p* < 0.001) and KYNA concentrations correlated positively with IIEF-5 scores. This finding suggests that a decrease in KYNA-mediated AhR signaling may be associated with hormonal alterations observed in ED.

The interplay between KYNA and QA is also noteworthy, as they act as a NMDA receptor antagonist and agonist respectively. NMDA receptor signalling has been implicated in the regulation of neuronal nitric oxide synthase (nNOS) [14]. In our study, KYNA levels were significantly lower in the ED group (1,39 ng/ml vs. 2,19 ng/ml, *p* < 0.001), while QA levels were elevated. Although lower KYNA might appear counterintuitive given its antagonistic role at the NMDA receptor, this shift may reflect an overall imbalance in the neurotoxic to neuroprotective branches of the kynurenine pathway [15]. Whether the observed KYNA/QA imbalance has functional consequences for NMDA-dependent NO signaling in erectile tissue warrants investigation through direct mechanistic studies.

In our study, 3-HK was found to be significantly higher in the ED group compared to controls and showed the highest exploratory performance in single-marker ROC analysis (apparent AUC 0.902). Literature reports that 3-HK and its metabolite 3-HAA contribute to the production of reactive oxygen species (ROS), which are associated with endothelial dysfunction and smooth muscle cell damage [16, 17]. A recent study also showed that there is a relationship between the syntax score indicating coronary atherosclerosis and 3-HK [18]. Taken together, these data suggest that elevated 3-HK levels may be associated with vascular damage in ED. Further functional studies targeting ROS levels, endothelial function and apoptotic pathways are needed to clarify this potential mechanistic link at the cellular level.

QA levels were found to be significantly higher in the ED group compared to controls (4.60 vs. 2.60 ng/mL, *p* < 0.001). However, in subgroup analysis, the distribution of QA was not linear: while no significant difference was observed between the control and mild ED groups, a significant increase was detected in moderate and severe cases (Table 2; Fig. 3). Post-hoc comparisons confirmed that QA concentrations were significantly higher in the moderate-to-severe spectrum than in the mild and control groups. Recent studies have reported that QA may contribute to ROS production through excitotoxicity and complexation with Fe²⁺ [4, 19], these mechanisms have been associated with NOS uncoupling and decreased NO bioavailability [20]. Whether QA accumulation is an active driver in the pathophysiology of ED or an epiphenomenon accompanying disease severity will be the focus of future mechanistic research.

## Conclusions

In conclusion, this study demonstrates a marked imbalance in kynurenine pathway in patients with ED: decreased TRP and KYNA levels alongside elevated concentrations of kynurenine, 3-HK, 3-HAA and QA. These findings suggest an association between ED and kynurenine pathway imbalance. However, as this is a cross-sectional study, whether these metabolic changes contribute to ED or accompany it remains to be clarified in future longitudinal studies. Our results underscore the role of tryptophan metabolism associated with ED and highlight the kynurenine pathway as a potential target for future diagnostic or therapeutic research.

### Limitations

This study has several important limitations. First, due to its cross-sectional design, a causal relationship between KP metabolites and ED cannot be established; it is not possible to determine whether the observed metabolic differences are the cause, consequence, or concomitant finding of ED. Second, the study was conducted at a single center. Third, since inflammatory markers such as IFN-γ and CRP and indicators of oxidative stress were not directly measured, the mechanistic pathways suggested in the discussion (IDO activation, ROS production, NOS uncoupling) should be considered as unconfirmed hypotheses. Fourth, lifestyle variables such as diet, physical activity, sleep quality, stress level and medication use, as well as penile Doppler ultrasonography findings, were not recorded; these factors are potential confounders that could affect both ED and tryptophan metabolism. Finally, the small sample size in some subgroups limits the statistical power of these subgroup comparisons. Although LOO analysis was utilized to assess the stability of statistical comparisons (Mann–Whitney U and Kruskal–Wallis tests, reporting minimum and maximum p-values) and leave-one-out cross-validation (LOOCV) was applied to evaluate the predictive performance of ROC analyses, external validation in an independent sample was not performed; therefore, the discriminant performance findings should be considered exploratory.

## Data Availability

The datasets generated and analyzed during the current study are available from the corresponding author on reasonable request.

## Abbreviations

ED: Erectile dysfunction
KP: Kynurenine pathway
TRP: Tryptophan
KYN: Kynurenine
KTR: Kynurenine-to-tryptophan ratio
3-HK: 3-hydroxykynurenine
3-HAA: 3-hydroxyanthranilic acid
KYNA: Kynurenic acid
QA: Quinolinic acid
IIEF-5: 5-item International Index of Erectile Function
IDO: Indoleamine 2,3-dioxygenase
KMO: Kynurenine 3-monooxygenase
AUC: Area under the curve
LC–MS/MS: Liquid chromatography–tandem mass spectrometry
VIF: Variance inflation factor
